# The Physician's Visiting List for 1874

**Published:** 1873-12

**Authors:** 


*The Physician's Visiting List* for 1874. Twenty Third Year of its Publication, Philadelphia: Lindsay & Blackiston.

Proves a useful list or appointment book for the dentist as well as for the medical practitioner. For a number of years we have been using this Visiting List as a dental appointment book, and would regret to be without it. It contains much useful information, such as Hall's Ready method in Asphyxia, Poisons and their Antidotes, Almanac, etc. etc.

